# Supplementary material for: Potential Stereoselective Binding of Trans-(±)-Kusunokinin and Cis-(±)-Kusunokinin Isomers to CSF1R
Source: Molecules. 2022 Jun 29;27(13):4194. doi: 10.3390/molecules27134194 (PMC9268608; doi:10.3390/molecules27134194)
Supplement: Supplementary file 1 [file molecules-27-04194-s001.zip › molecules-1780008-supplementary.pdf]

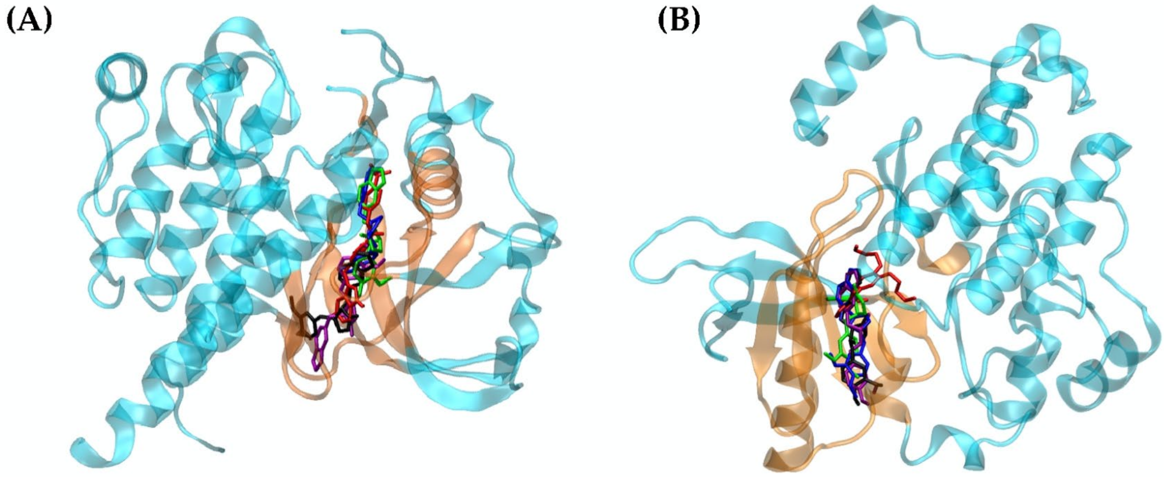

Figure S1. Selected candidate proteins binding pocket alignment. (**A**) The docked ligands in the CSF1R pocket were shown. (**B)** The docked ligands in the EGFR pocket were shown.

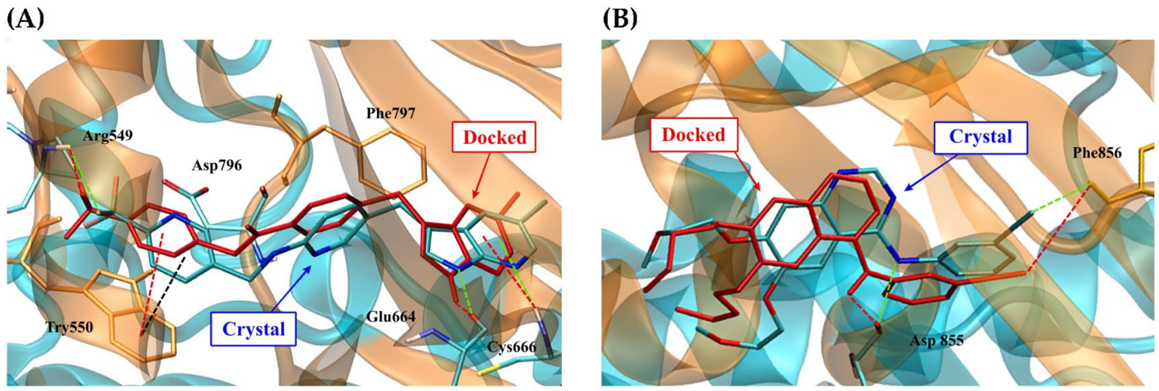

Figure S2. Alignment of docking structure to the reported crystal structure. (**A**) The docked poses of pexidartinib-CSF1R and (**B**) erlotinib-EGFR were illustrated.

Table S1. PDB identification codes of CSF1R-derived cancer progression associated proteins and Pubchem CIDs of selected known inhibitors.

Target name	Known inhibitor name	PubChem CID
	**Anti-apoptosis and Survival**	
COX2	Sorafenib	216239
Hsp70	Apoptozole	24894064
Hsp90a	Pu-H54	136236968
Hsp90b	EC44	11517212
PAK6	Sunitinib	5329102
Survivin	YM155	11178236
XIAP	A4E	134611691
	**Cell growth and Proliferation**	
cMyc	MYCi975	139600320
ER	E4D	448577

PLK1	Pyrimidodiazepinone	66746050
ACD10	SQ-22536	5270
AR	(*R*)-Bicalutamide	56069
cdc25A	Quinonoid	136254585
cdc25B	Quinonoid	136254585
CDK1	CGP74514A	2794188
CDK2	Dinaciclib	46926350
CDK4	Palbociclib	5330286
cyclinA	ligand 107	1712
cyclinB1	Q27097368	24864079
cyclinD1	Fascaplysin	73292
GSK3b	Tideglusib	11313622
mTOR	AZD8055	25262965
PKA	AT13148	24905401
PKC	Enzastaurin	176167
ragC	Palomid-529	11998575
CSF1R	Pexidartinib	25151352
EGFR	Erlotinib	176870
FLT3	Gilteritinib	49803313
IL6R	Terminolic acid	12314613
	**Metastasis**	
cFos	T-5224	23626877
cJun	T-5224	23626877
mmp12	BAY-7598	118425786
mmp9	ARP101	11292680
snail	Chembl4517265	46218884
AKR1B1	Epalrestat	1549120
ALP	Levamisole	26879
PALP	Levamisole	26879

Table S1. *Cont.*

Target name	Known inhibitor name	PubChem CID
TGFB1	SB431542	4521392
TGFB2	J2V	121411874
	**Signaling molecules**	
AKT	Capivasertib	25227436
CRAF	Sorafenib	216239
ERK1	SCH772984	24866313
ERK2	Q27455064	91819626
Grb2	CGP-78850	9940118
IKK	Dehydrocostus Lactone	73174
JAK1	CHEMBL3779913 (66P)	135567142
JAK2	NVP-BSK805	131648244
JAK3	inhibitor 6 (7KX)	137348637
KRAS	Sotorasib	137278711
MEK1	Trametinib	11707110
MEK2	Trametinib	11707110
MNK2	CHEMBL4457368 (BV9)	145915878
p38	PD169316	4712
PI3K	Apitolisib	25254071
PIM1	6SD	2756469
RAC1	NSC-23766	409805
SMAD3	SIS3	16079005
STAT1	Fludarabine	657237
STAT3	STAT3-IN-3	138454793
STAT5	STAT5-IN-2	137628635

Table S2. One-way ANOVA of four kusunokinin isomers docking score to the same protein.

Target name	Kusunokinin isomer docking score				Known inhibitor		One-Way ANOVA			
	Trans-(-)	*Trans*-(+)	*Cis*-(-)	*Cis*-(+)	Name	Docking score	Count	Sum	Average	Variance
**Anti-apoptosis and Survival**										
COX2[1]	−10.60	−9.68	−9.62	−10.57	Sorafenib	−9.06	4	−40.47	−10.1175	0.292158333
Hsp70	−8.93	−7.84	−8.43	−9.03	Apoptozole	−8.24	4	−34.23	−8.5575	0.297691667
Hsp90a	−10.60	−10.09	−10.43	−10.77	Pu-H54	−9.43	4	−41.89	−10.4725	0.084291667
Hsp90b	−10.80	−9.28	−10.63	−9.74	EC44	−10.20	4	−40.45	−10.1125	0.524091667
PAK6	−8.55	−7.56	−8.09	−8.39	Sunitinib	−7.50	4	−32.59	−8.1475	0.189758333
survivin	−8.78	−8.63	−8.76	−8.13	YM155	−7.25	4	−34.3	−8.575	0.092433333
XIAP	−7.67	−6.74	−7.70	−7.49	A4E	−10.66	4	−29.6	−7.4	0.2022
**Cell Growth and Proliferation**										
cMyc	−7.30	−6.32	−7.22	−6.55	MYCi975	−6.19	4	−27.39	−6.8475	0.236758333
ER	−8.87	−8.43	−9.02	−8.87	E4D	−11.74	4	−35.28	−8.82	0.0714
PLK1	−8.87	−8.87	−8.92	−8.84	Pyrimidodiazepinone	−5.92	4	−35.5	−8.875	0.0011
ACD10	−12.59	−11.32	−12.21	−12.12	SQ−22536	−8.23	4	−48.24	−12.06	0.284866667
AR	−8.50	−8.35	−9.36	−9.03	(*R*)-Bicalutamide	−8.55	4	−35.24	−8.81	0.219533333
cdc25A	−9.08	−7.66	−8.48	−9.15	Quinonoid	−6.90	4	−34.37	−8.5925	0.476891667
cdc25B	−7.62	−7.46	−7.63	−7.78	Quinonoid	−6.91	4	−30.49	−7.6225	0.017091667
CDK1	−10.34	−9.27	−9.79	−9.69	CGP74514A	−10.96	4	−39.09	−9.7725	0.193891667
CDK2	−9.74	−9.14	−9.90	−9.90	Dinaciclib	−8.93	4	−38.68	−9.67	0.130533333
CDK4	−8.16	−7.22	−7.32	−7.55	Palbociclib	−9.03	4	−30.25	−7.5625	0.177758333
cyclinA	−8.89	−9.56	−8.92	−8.69	ligand 107	−9.76	4	−36.06	−9.015	0.142433333
cyclinB1	−10.45	−10.08	−10.24	−10.84	Q27097368	−9.40	4	−41.61	−10.4025	0.108025
cyclinD1	−8.18	−7.04	−8.20	−8.42	Fascaplysin	−7.57	4	−31.84	−7.96	0.388
GSK3b	−10.64	−10.01	−9.91	−10.05	Tideglusib	−8.59	4	−40.61	−10.1525	0.109091667
mTOR	−9.12	−8.46	−9.09	−8.95	AZD8055	−10.21	4	−35.62	−8.905	0.0935
PKA	−9.48	−8.46	−9.58	−9.61	AT13148	−12.83	4	−37.13	−9.2825	0.303758333
PKC	−9.83	−8.99	−9.87	−9.79	Enzastaurin	−12.69	4	−38.48	−9.62	0.177466667
ragC	−9.21	−8.73	−8.95	−8.82	Palomid-529	−8.85	4	−35.71	−8.9275	0.043625
CSF1R	−11.84	−9.29	−10.53	−10.40	Pexidartinib	−11.59	4	−42.06	−10.515	1.0899
EGFR	−9.82	−9.45	−9.45	−9.50	Erlotinib	−8.82	4	−38.22	−9.555	0.031766667
FLT3	−9.24	−9.05	−9.22	−8.89	Gilteritinib	−9.64	4	−36.4	−9.1	0.026866667
IL6R	−7.74	−7.48	−7.61	−7.73	Terminolic acid	−7.11	4	−30.56	−7.64	0.014866667
**Metastasis**										
cFos	−5.63	−5.07	−5.32	−5.51	T-5224	−6.20	4	−21.53	−5.3825	0.059691667
cJun	−5.66	−4.95	−5.27	−5.51	T-5224	−7.72	4	−21.39	−5.3475	0.096025
mmp12	−11.19	−10.23	−10.71	−10.80	BAY-7598	−12.47	4	−42.93	−10.7325	0.155625
mmp9	−10.41	−10.48	−10.37	−9.64	ARP101	−12.27	4	−40.90	−10.225	0.154166667
snail	−7.85	−7.53	−7.49	−7.47	Chembl4517265	−7.70	4	−30.34	−7.585	0.031833333
AKR1B1	−11.32	−11.15	−11.46	−11.59	Epalrestat	−10.14	4	−45.52	−11.38	0.035666667
ALP	−7.26	−7.95	−7.36	−7.34	Levamisole	−6.08	4	−29.91	−7.4775	0.10109167
PALP	−6.92	−6.77	−7.69	−7.48	Levamisole	−5.79	4	−28.86	−7.215	0.19363333
TGFB1	−7.08	−6.92	−7.06	−7.62	SB431542	−7.17	4	−28.68	−7.17	0.095066667
TGFB2	−7.96	−7.73	−7.88	−8.21	J2V	−7.10	4	−31.78	−7.945	0.0403
**Signaling molecules**										
AKT	−9.64	−8.97	−9.12	−9.23	Capivasertib	−8.50	4	−36.96	−9.24	0.082466667
CRAF	−8.60	−8.11	−9.14	−8.82	Sorafenib	−9.06	4	−34.67	−8.6675	0.187291667
ERK1	−9.63	−9.24	−9.54	−9.65	SCH772984	−12.96	4	−38.06	−9.515	0.0359
ERK2	−9.15	−8.65	−9.11	−9.42	Q27455064	−8.39	4	−36.33	−9.0825	0.102091667
Grb2	−8.03	−7.32	−7.78	−7.92	CGP-78850	−7.88	4	−31.05	−7.7625	0.097491667
IKK	−7.89	−7.70	−7.80	−8.18	Dehydrocostus Lactone	−7.28	4	−31.57	−7.8925	0.042758333
JAK1	−8.64	−8.56	−8.60	−8.44	66P	−10.52	4	−34.24	−8.56	0.007466667
JAK2	−8.20	−7.66	−8.40	−8.17	NVP-BSK805	−11.22	4	−32.43	−8.1075	0.099425
JAK3	−8.97	−8.62	−9.03	−8.81	inhibitor 6 (7KX)	−7.30	4	−35.43	−8.8575	0.033691667
K-RAS	−7.55	−7.19	−7.85	−7.69	Sotorasib	−7.11	4	−30.28	−7.57	0.0792
MEK1	−10.34	−9.48	−10.63	−10.14	Trametinib	−13.05	4	−40.59	−10.1475	0.238491667
MEK2	−10.17	−9.41	−10.95	−10.06	Trametinib	−12.17	4	−40.59	−10.1475	0.398691667
MNK2	−9.86	−9.60	−10.02	−9.94	BV9	−8.64	4	−39.42	−9.855	0.033166667

Table S2. *Cont.*

Target name	Kusunokinin isomer docking score				Known inhibitor		One-Way ANOVA			
	Trans-(-)	*Trans*-(+)	*Cis*-(-)	*Cis*-(+)	Name	Docking score	Count	Sum	Average	Variance
p38	−8.73	−7.89	−8.63	−8.61	PD169316	−7.94	4	−33.86	−8.465	0.1497
PI3K	−9.47	−8.66	−9.12	−9.06	Apitolisib	−9.55	4	−36.31	−9.0775	0.110158333
PIM1	−9.38	−8.03	−9.16	−9.30	6SD	−6.95	4	−35.87	−8.9675	0.398891667
RAC1	−10.12	−9.33	−9.36	−10.12	NSC-23766	−6.94	4	−38.93	−9.7325	0.200358333
SMAD3	−8.33	−7.78	−8.06	−7.91	SIS3	−9.05	4	−32.08	−8.02	0.0558
STAT1	−7.41	−7.75	−7.49	−7.37	Fludarabine	−5.41	4	−30.02	−7.505	0.029166667
STAT3	−7.84	−7.51	−7.63	−7.82	STAT3-IN-3	−9.87	4	−30.80	−7.7	0.025
STAT5	−9.52	−9.27	−8.78	−8.96	IN-2	−8.45	4	−36.53	−9.1325	0.107691667

[1]Black letters represent 12 candidate proteins that passed both criteria for a potential inhibition.

Table S3. Reference- and MD rearranged-residue number of CSF1R kinase domain.

Reference residue number	MD residue number	Amino acid abbreviates	Reference residue number	MD residue number	Amino acid abbreviates
544	1	PRO	594	46	GLY
545	2	LYS	595	47	LYS
546	3	TYR	596	48	VAL
547	4	GLN	597	49	VAL
548	5	VAL	598	50	GLU
549	6	ARG	599	51	ALA
550	7	TRP	600	52	THR
551	8	LYS	601	53	ALA
552	9	ILE	602	54	PHE
553	10	ILE	603	55	GLY
554	11	GLU	604	56	LEU
555	12	SER	605	57	GLY
561	13	TYR	606	58	LYS
562	14	THR	607	59	GLU
563	15	PHE	608	60	ASP
564	16	ILE	609	61	ALA
565	17	ASP	610	62	VAL
566	18	PRO	611	63	LEU
567	19	THR	612	64	LYS
568	20	GLN	613	65	VAL
569	21	LEU	614	66	ALA
570	22	PRO	615	67	VAL
571	23	TYR	616	68	LYS
572	24	ASN	617	69	MET
573	25	GLU	618	70	LEU
574	26	LYS	619	71	LYS
575	27	TRP	620	72	SER
576	28	GLU	621	73	THR
577	29	PHE	622	74	ALA
578	30	PRO	623	75	HIS
579	31	ARG	624	76	ALA
580	32	ASN	625	77	ASP
581	33	ASN	626	78	GLU
582	34	LEU	627	79	LYS
583	35	GLN	628	80	GLU
584	36	PHE	629	81	ALA
585	37	GLY	630	82	LEU
586	38	LYS	631	83	MET
587	39	THR	632	84	SER
588	40	LEU	633	85	GLU

589	41	GLY	634	86	LEU
590	42	ALA	635	87	LYS
591	43	GLY	636	88	ILE
592	44	ALA	637	89	MET
593	45	PHE	638	90	SER

Table S3. *Cont.*

Reference residue number	MD residue number	Amino acid abbreviates	Reference residue number	MD residue number	Amino acid abbreviates
639	91	HIS	665	117	TYR
640	92	LEU	666	118	CYS
641	93	GLY	667	119	THR
642	94	GLN	668	120	TYR
643	95	HIS	669	121	GLY
644	96	GLU	670	122	ASP
645	97	ASN	671	123	LEU
646	98	ILE	672	124	LEU
647	99	VAL	673	125	ASN
648	100	ASN	674	126	PHE
649	101	LEU	675	127	LEU
650	102	LEU	676	128	ARG
651	103	GLY	677	129	ARG
652	104	ALA	678	130	LYS
653	105	CYS	679	131	ALA
654	106	THR	680	132	GLU
655	107	HIS	681	133	ALA
656	108	GLY	682	134	MET
657	109	GLY	683	135	LEU
658	110	PRO	684	136	GLY
659	111	VAL	665	117	TYR
660	112	LEU	666	118	CYS
661	113	VAL	667	119	THR
662	114	ILE	668	120	TYR
663	115	THR	669	121	GLY
664	116	GLU	670	122	ASP
665	117	TYR	671	123	LEU
666	118	CYS	672	124	LEU
667	119	THR	673	125	ASN
668	120	TYR	674	126	PHE
669	121	GLY	675	127	LEU
670	122	ASP	676	128	ARG
671	123	LEU	677	129	ARG
672	124	LEU	678	130	LYS
673	125	ASN	679	131	ALA
674	126	PHE	680	132	GLU
675	127	LEU	681	133	ALA
676	128	ARG	682	134	MET
677	129	ARG	683	135	LEU
678	130	LYS	684	136	GLY
679	131	ALA	668	120	TYR
680	132	GLU	669	121	GLY
662	114	ILE	670	122	ASP
663	115	THR	671	123	LEU
664	116	GLU	672	124	LEU

Table S3. *Cont.*

Reference residue number	MD residue number	Amino acid abbreviates	Reference residue number	MD residue number	Amino acid abbreviates
673	125	ASN	765	158	GLY
674	126	PHE	766	159	MET
675	127	LEU	767	160	ALA
676	128	ARG	768	161	PHE
677	129	ARG	769	162	LEU
678	130	LYS	770	163	ALA
679	131	ALA	771	164	SER
680	132	GLU	772	165	LYS
681	133	ALA	773	166	ASN
682	134	MET	774	167	CYS
683	135	LEU	775	168	ILE
684	136	GLY	776	169	HIS
673	125	ASN	777	170	ARG
674	126	PHE	778	171	ASP
675	127	LEU	779	172	VAL
676	128	ARG	780	173	ALA
677	129	ARG	781	174	ALA
678	130	LYS	782	175	ARG
679	131	ALA	783	176	ASN
680	132	GLU	784	177	VAL
681	133	ALA	785	178	LEU
682	134	MET	786	179	LEU
683	135	LEU	787	180	THR
684	136	GLY	788	181	ASN
685	137	PRO	789	182	GLY
686	138	SER	790	183	HIS
687	139	LEU	791	184	VAL
747	140	GLY	792	185	ALA
748	141	ARG	793	186	LYS
749	142	PRO	794	187	ILE
750	143	LEU	795	188	GLY
751	144	GLU	796	189	ASP
752	145	LEU	797	190	PHE
753	146	ARG	798	191	GLY
754	147	ASP	799	192	LEU
755	148	LEU	800	193	ALA
756	149	LEU	801	194	ARG
757	150	HIS	802	195	ASP
758	151	PHE	803	196	ILE
759	152	SER	804	197	MET
760	153	SER	805	198	ASN
761	154	GLN	806	199	ASP
762	155	VAL	807	200	SER
763	156	ALA	808	201	ASN
764	157	GLN	809	202	TYR

Table S3. *Cont.*

Reference residue number	MD residue number	Amino acid abbreviates	Reference residue number	MD residue number	Amino acid abbreviates
810	203	ILE	844	237	LEU
811	204	VAL	845	238	LEU
812	205	LYS	846	239	TRP
813	206	GLY	847	240	GLU
814	207	ASN	848	241	ILE
815	208	ALA	849	242	PHE
816	209	ARG	850	243	SER
817	210	LEU	851	244	LEU
818	211	PRO	852	245	GLY
819	212	VAL	853	246	LEU
820	213	LYS	852	245	GLY
821	214	TRP	853	246	LEU
822	215	MET	848	241	ILE
823	216	ALA	849	242	PHE
824	217	PRO	850	243	SER
825	218	GLU	851	244	LEU
826	219	SER	854	247	ASN
827	220	ILE	855	248	PRO
828	221	PHE	856	249	TYR
829	222	ASP	857	250	PRO
830	223	SER	858	251	GLY
831	224	VAL	859	252	ILE
832	225	TYR	860	253	LEU
833	226	THR	861	254	VAL
834	227	VAL	862	255	ASN
835	228	GLN	863	256	SER
836	229	SER	864	257	LYS
837	230	ASP	865	258	PHE
838	231	VAL	866	259	TYR
839	232	TRP	867	260	LYS
840	233	SER	868	261	LEU
841	234	TYR	869	262	VAL
842	235	GLY	870	263	LYS
843	236	ILE	871	264	ASP
844	237	LEU	872	265	GLY
845	238	LEU	873	266	TYR
846	239	TRP	874	267	GLN
847	240	GLU	875	268	MET
848	241	ILE	876	269	ALA
849	242	PHE	877	270	GLN
850	243	SER	878	271	PRO
851	244	LEU	879	272	ALA
841	234	TYR	880	273	PHE
842	235	GLY	881	274	ALA
843	236	ILE	882	275	PRO

Table S3. *Cont.*

Reference residue number	MD residue number	Amino acid abbreviates	Reference residue number	MD residue number	Amino acid abbreviates
883	276	LYS	892	285	CYS
884	277	ASN	893	286	TRP
885	278	ILE	894	287	ALA
886	279	TYR	895	288	LEU
887	280	SER	896	289	GLU
888	281	ILE	897	290	PRO
889	282	MET	898	291	THR
890	283	GLN	899	292	HIS
891	284	ALA	900	293	ARG
892	285	CYS	901	294	PRO
893	286	TRP	902	295	THR
894	287	ALA	903	296	PHE
895	288	LEU	904	297	GLN
896	289	GLU	905	298	GLN
897	290	PRO	906	299	ILE
883	276	LYS	907	300	THR
884	277	ASN	908	301	SER
885	278	ILE	909	302	PHE
886	279	TYR	910	303	LEU
887	280	SER	911	304	GLN
888	281	ILE	912	305	GLU
889	282	MET	913	306	GLN
890	283	GLN	914	307	ALA
891	284	ALA	915	308	GLN
